# Inducible nitric oxide synthase expression in gastric adenocarcinoma: impact on lymphangiogenesis and lymphatic metastasis

**DOI:** 10.1186/1746-1596-8-151

**Published:** 2013-09-17

**Authors:** Nimet Karadayı, Nilufer Onak Kandemır, Dilek Yavuzer, Taner Korkmaz, Gonca Gecmen, Furuzan Kokturk

**Affiliations:** 1 Department of Pathology, Dr. Lutfi Kirdar Training and Research Hospital, Istanbul, Turkey; 2 Department of Pathology, Faculty of Medicine, Bulent Ecevit University, Zonguldak, Turkey; 3 Department of Medical Oncology, Dr. Lutfi Kirdar Training and Research Hospital, Istanbul, Turkey; 4 Department of Biostatistics, Faculty of Medicine, Bulent Ecevit University, Zonguldak, Turkey

**Keywords:** Gastric carcinoma, Inducible nitric oxide synthases, Lymphangiogenesis, Lymph node metastasis

## Abstract

**Background:**

Lymphatic metastasis is the most important parameter in the spread of gastric carcinomas. Nitric oxide (NO) is a signaling molecule that plays an important role in inflammation and carcinogenesis. In this study, the possible link between inducible nitric oxide synthase (iNOS) expression with lymphangiogenesis and the clinicopathological parameters of gastric carcinomas was investigated.

**Methods:**

In this study, iNOS expression and D2-40 (lymphatic endothelium-specific marker monoclonal antibody) reactivity were examined immunohistochemically in 41 gastric adenocarcinoma and 20 non-neoplastic gastric tissues. iNOS expression was scored semiquantitatively in the tumor parenchyma and stroma. D2-40-positive lymphatic vessels were used in the determination of lymphatic invasion and intratumoral and peritumoral lymphatic vascular density.

**Results:**

iNOS expression was higher in gastric carcinoma tissue compared with non-neoplastic tissue. Particularly, iNOS expression in tumor cells was found to be closely related to lymphangiogenesis and lymphatic metastasis. The density of lymphatic invasion as well as intratumoral and peritumoral lymphatic vascular density were positively correlated with lymph node metastasis.

**Conclusions:**

Our results suggest that iNOS-mediated NO formation plays an important role in gastric carcinogenesis, tumor lymphangiogenesis, and the development of lymphatic metastases. Inhibition of the NO pathway may be an alternative treatment of gastric carcinomas.

**Virtual slides:**

The virtual slides for this article can be found here: http://www.diagnosticpathology.diagnomx.eu/vs/1713572940104388.

## Background

Solid tumor growth depends on the proliferative activity of the tumor as well as tumor angiogenesis [1]. The most important factors in the initiation and progression of angiogenesis are nitric oxide (NO) and the vascular endothelial growth factor (VEGF) family. NO exists in various cell types and is synthesized from L-arginine by the nitric oxide synthase (NOS) enzyme family. Inducible nitric oxide synthase (iNOS) is the most-active NOS isoform [2-4].

In recent years, studies have shown that NO plays an important role in tumor lymphangiogenesis and lymphatic metastasis. NO, which is synthesized by lymphatic endothelial cells (LECs), regulates lymphatic permeability and lymphatic flow. Increased vascular permeability and vasodilatation by NO facilitates the spread of tumor cells via lymphovascular invasion (LVI). Additionally, NO is thought to accelerate lymphangiogenesis via increasing the expression of VEGF-C/VEGF-D and VEGF-R2/VEGF-R3 [5-7]. Studies of head and neck squamous cell carcinoma have shown that the NOS activity of tumor cells is closely associated with lymphangiogenesis and lymph node metastasis [8].

As with other solid tumors, lymphatic invasion (LI) and lymph node metastasis are among the most important steps of gastric carcinoma progression. However, in the literature, few studies have investigated the effect of NOS expression on lymphangiogenesis and lymphatic metastasis in gastric cancer [5,9-11]. In this study, the effect of iNOS expression on tumor lymphangiogenesis was investigated in gastric adenocarcinoma. In addition, we examined the relationship between iNOS expression and other clinicopathological prognostic parameters, primarily LI and lymph node metastasis.

## Methods

### Patients

#### Gastric carcinoma cases

41 patients with gastric adenocarcinoma who consecutively underwent surgical resection between 01. 01. 2007 and 30. 12. 2010 were investigated in this study. Clinical parameters were obtained by review of surgical pathology reports, and the Oncology Data Bank. Patients undergoing neoadjuvant chemotherapy and/or radiotherapy were excluded. Surgical margins were confirmed negative for all cases included in this study. They had no detectable metastasis in liver, peritoneum, and distant organ at the time of surgery. No other previous or concomitant primary cancer was present. The total number of dissected lymph nodes of the 41 gastric carcinoma patients was 1021, with an average of 18.8 ±11.3 (range 15–78) dissected nodes per case. Seventeen (41.5%) of the cases displayed weight loss, 18 (43.9%) of the cases diplayed other gastrointestinal symptoms (e.g. stomachache, vomiting, indigestion) and 6 (14.6%) presented anemia with hemoglobin (HGB) < 90 g/l. 53.7% (n = 22) of the tumors were in antrum, 14.6% (n = 6) of these in corpus, and 31.7% (n = 13) of these in cardia. The median follow-up time of the patients was 16 month and ranged from 11 to 65 month.

Investigation of gastric carcinoma cases with respect to conditions accompanying the tumor revealed that 21 (51.2%) patients had Helicobacter Pylori-associated gastritis, 9 (22%) had intestinal metaplasia, 5 (12.2%) had chronic atrophic gastritis, 4 (9.7%) had intraepithelial neoplasia (dysplasia), and 2 (4.9%) had non-neoplastic polyps. None of the cases had a family history of “Hereditary diffuse gastric cancer” or a genetically proven mutation.

#### Non-neoplastic gastric tissue

This study included non-neoplastic gastric tissues of 20 patients who underwent sleeve gastrectomy for obesity (9 men, 11 women, ranging in age from 25 to 42 years, mean 32 ± 9.2 years).

The study was performed in accordance with the Helsinki Declaration, internationally recognized guidelines, and the privacy of patients was protected by decoding of data, according to the privacy regulations of the Dr. Lutfi Kirdar Training and Research Hospital (Istanbul, Turkey).Written informed consent was obtained from the patient and the protocol was approved by the Institutional Review Board.

The study was performed in accordance with the Helsinki Declaration, and the privacy of patients was protected by decoding of data, according to the privacy regulations of the Dr. Lutfi Kirdar Training and Research Hospital (İstanbul, Turkey).

### Pathological evaluation

Gross types of tumors was carried out according to Borrmann classification. The histopathological diagnoses, histological grading, tumour stage, histological type, Lauren classification, LVI, and local inflammatory response were confirmed through the examination of archived hematoxylin and eosin (H&E) slices. Tumor-node-metastasis (TNM) staging was carried out according to the American Joint Committee on Cancer (AJCC) classification [12,13], and histological grading was performed according to World Health Organisation (WHO) criteria [14]. Only patients with histologically typed adenocarcinoma [not otherwise specified (NOS), papillary, tubular, mucinous, poorly cohesive carcinoma (including signet ring cell carcinoma and other variants)] tumors were included in the sample [14,15]. LVI and LI in the tumor tissue were evaluated according to previously defined criteria [16].

Local inflammatory response in the tumor tissue was evaluated according to the criteria defined by Klintrup et al. [17]. Tumours were scored based on the appearance at the deepest area of tumour invasion on a four-point score. A score of 0 indicated that there was no increase in the inflammatory cells at the deepest point of the tumours invasive margin; score 1 denoted a mild and patchy increase in the inflammatory cells; score 2 denoted a prominent inflammatory reaction forming a continuous band at the invasive margin with some evidence of destruction of cancer cell islands and score 3 denoted a florid ‘cup-like’ inflammatory infiltrate at the invasive edge with frequent destruction of cancer cell islands. All specimens (tumours with 5 mm of adjacent non-cancerous tissues) were collected and embedded in paraffin for immunohistochemical study.

### Immunohistochemistry

All tissues were fixed in 10% neutral buffered formalin and embedded in paraffin using standard surgical pathology protocols. Tissue sections (4 μm) were dewaxed and antigen retrieval was performed in citrate buffer (pH = 6) for 5 min using a microwave oven. Immunohistochemical staining procedures were performed in line with routine surgical pathology protocols. The following antibodies were used in immunohistochemical studies: iNOS (clone [M-19]-G, 1:100 dilution; Santa Cruz, Heidelberg, Germany) and D2-40 antigen (clone D2-40; 1:50 dilution; Signet Laboratories, Dedham, MA, USA). Immunohistochemical reactions were developed with diaminobenzidine as the chromogenic peroxidase substrate. The sections were counterstained with hematoxylin after immunohistochemistry [6,8].

Specificity was verified by negative control reactions without primary mAbs and by the appropriate reaction for each antigen in positive control tissues. The immunohistochemically stained sections were reviewed independently by two pathologists (NK, NOK) without knowledge of the patients’clinicopathological details. When disagreement arose, the slides in question were jointly reviewed.

#### Assessment of immunohistochemical stain of iNOS

iNOS expression in pathology specimens was analyzed in 3 different localizations: tumor cells (iNOS-T), endothelial cells (iNOS-E), and tumor-associated stromal fibroblasts (iNOS-F). To score the iNOS staining pattern, only cells with evidence of cytoplasmic, perinuclear and luminal staining were considered positive [5,7,9-11,18-20]. The intensity of immunostainings with iNOS antibodies was evaluated by dividing the staining reaction into four scores: 1 = weak, 2 = moderate, 3 = strong, and 4 = very strong staining intensity. The quantity of the immunostaining was scored as follows; 0 = no positive immunostaining, 1 = <25%, 2 = 25–49%, 3 = 50–75%, and 4= >75% of cells showing immuno-positivity. By adding up the qualitative and quantitative scores, a sum score was obtained, which was then divided into five main groups: - (score 0) = no immunostaining; + (score 1–2) = weak immunostaining; ++ (score 3–4) = moderate immunostaining; +++ (score 5–6) = strong immunostaining; ++++ (score 7–8) = very strong immunostaining [18-20].

#### Assessment of immunohistochemical stain of D2-40: Lymphatic invasion (LI), lymphatic invasion density (LID [nLI/mm2]), and lymphatic vascular density (LVD)

In order to determine LI, LID [nLI/mm2], and LVD, LECs spesific D2-40 mAb was used. Non-neoplastic tumor-adjacent lymphatic endothelial cells were used as internal positive controls for D2-40. LI of the tumor was established when at least one neoplastic cell cluster was clearly visible inside a D2-40 positive lymph vessel, following Yamauchi et al. [16]. Total LI number (nLI) was determined for each case as whole slide being treated with D2-40 immunohistochemically. Tissue areas in sections D2-40 was applied were measured using digital planimeter (Visitrak, Smith&- Nephew, 00 00 00 42 17) in each case as previously described [21]. LI number in a mm2 was determined via dividing nLI in each case by tissue area. Total tissue area measured in gastric carcinoma cases is 9664 mm2 (mean ± SD; 235.71 ± 57.38, range 133–376).

The method of quantifying LVD has been reported previously [19-21]. The D2-40 stained sections were first scanned at low magnification (100×) to select ten “hotspots” (areas with the greatest amount of distinct brown staining) in each tumor [22-24]. These hotspots were then counted at 400× with a microscope ocular grid corresponding to an examination area of 0.1885 mm2 (i.e., 40 × objective lens and 10× ocular lens; 0.1885 mm2 per field). Any immunostained cells or separate clusters of endothelial cells, with or without an identifiable lumen, were considered and counted as a single vessel. Depending on the size of the hot spot, 1 to 3 readings were taken. In the absence of apparent hot spots, 10 randomly selected areas were counted. The LVD for each case was expressed by the mean value (total number of vessels in 10 hot spot microscopic fields/10). For each case, the peritumoral LVD (P-LVD) [hot spots selected in the peripheral tissues within 2 mm of the tumour margins] and intratumoral LVD (I- LVD) [hot spots located within the tumour and surrounded by tumor cells] were calculated separately [22-24].

### Statistical analysis

Statistical analyses were performed with SPSS 18.0 software (SPSS Inc., Chicago, IL, USA). Distribution of data was determined by Shapiro-Wilks test. Continuous variables were expressed as mean ± std. deviation, categorical variables as frequency and percent. Continuous variables were compared with independent sample *t* test or the Mann–Whitney *U* test for two group. The one way analysis of variance (ANOVA) or Kruskal-Wallis test was used to determine for differences between three or more groups. The Tukey test was used as a post hoc test, if the Anova test is statistically significant. Bonferroni adjusted Mann–Whitney *U* test was used for post-hoc test after Kruskal-Wallis test. Pearson’s or Spearman’s correlation analysis was performed to determine the relationship between continious variables. p value of less than 0.05 was considered statistically significant for all tests.

## Results

### Clinical and pathological findings

Clinicopathological parameters are summarized in Table 1.

**Table 1:** Clinicopathologic characteristics of gastric carcinoma cases

**Clinicopathologic variables**	**Number of cases *(n)***	**Percent *(%)***
Age (years)	62.00 ± 10.94 (range: 35–82)	
Tumor size (cm)	6.46 ± 2.66 (range: 2–13)	
Gender		
Male	26	63.4
Female	15	36.6
Location of tumor		
Cardia	13	31.7
Corpus	6	14.6
Antrum	22	53.7
Borrmann type		
I	0	0
II	6	14.6
III	26	63.4
IV	9	22
Lauren classificafion		
Intestinal	19	46.3
Diffuse	7	17.1
Mixed	15	36.6
Histological type		
NOS/tubular/papillary	31	75.6
Mucinous/poorly cohesive	10	24.4
Differentiation		
Well/moderate	20	48.7
Poorly/signet ring cell	21	51.3
Nodal status		
pN0	9	22
pN1	7	17.1
pN2	12	29.2
pN3	13	31.7
Depth of invasion		
pT1	2	4.9
pT2	2	4.9
pT3	25	61
pT4	12	29.2
Stage (TNM)		
I	3	7.3
II	16	39.1
III	22	53.6
IV	0	0
Inflammatory response		
Score 0	0	0
Score 1	11	26.8
Score 2	12	29.3
Score 3	18	43.9
Lymphovascular invasion [H&E]		
Positive	31	75.6
Negative	10	24.4
Lymphatic invasion [D2-40]		
Positive	35	85.4
Negative	6	14.6

### Immunohistochemical findings

#### iNOS

##### Gastric carcinoma cases

**Figure 1:**
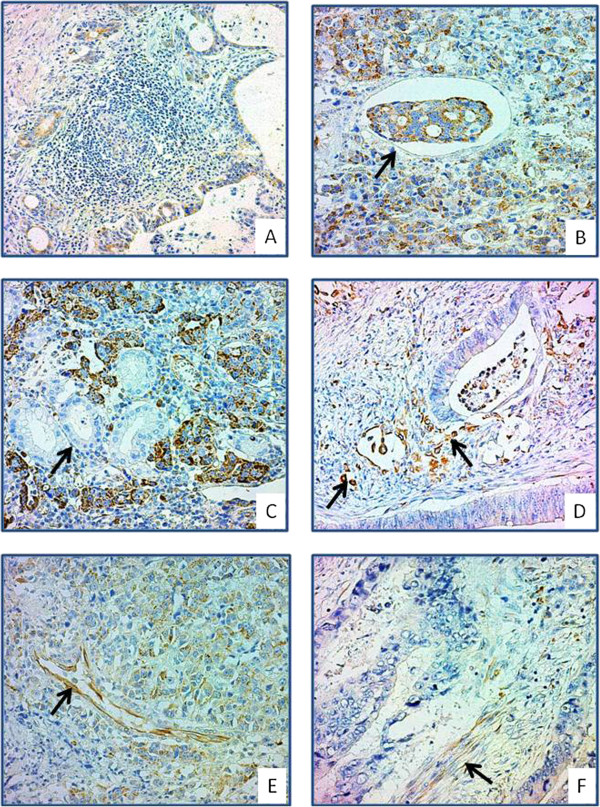
**iNOS immunoreactivity in gastric carcinoma tissue. (A)** A focal/weak/luminal immunoreaction is observed in a well-differentiated adenocarcinoma case. **(B)** Intense/diffuse/cytoplasmic iNOS immunoreactivity is observed in a poorly differentiated tumor tissue. **(C)** No iNOS expression was detected in non-neoplastic glands in the same case. **(D)** Despite focal/weak iNOS immunoreactivity in well-differentiated tumor areas, there is strong iNOS expression in tumor cells of the invasive phenotype. iNOS immunoreactivity is demonstrated in endothelial cells **(E)** and fibroblasts **(F)** in the tumor stroma.

Analysis of iNOS expression in tumor cells revealed that five (12.2%) cases showed no iNOS expression (Score 0). Thirty-six (87.8%) cases had varying degrees of iNOS expression [iNOS-T score (mean ± standard deviation): 4.05 ± 2.46; range: 1–8]. iNOS immunoreactivity was heterogeneous among the different types of tumors and/or in the same tumor. Although weak/focal/luminal staining (score 1–2) was frequently present at well-differentiated tumor sites, a strong/homogeneous/cytoplasmic reaction (score 7–8) was evident at poorly differentiated tumor sites (Figure 1A-B). None of the gastric carcinoma cases displayed iNOS expression in the non-neoplastic epithelium (Figure 1C). Seven (17.1%) cases had a strong iNOS immunoreaction in invasive tumor cells that had lost their polarities and adhesions [epithelial–mesenchymal transition (EMT)-like dedifferentiation] (Figure 1D) [24]. In all the gastric carcinoma samples, varying degrees of iNOS expression were detected in endothelial cells (iNOS-E score: 5.88 ± 2.02; range 2–8) (Figure 1E) and tumor-associated stromal fibroblasts (iNOS-F score: 4.71 ± 1.61; range 2–8) (Figure 1F).

The iNOS scores of all cellular components of gastric carcinoma cases are summarized in Table 2.

**Table 2:** Distribution of iNOS scores of tumor cells (iNOS-T), tumor-associated stromal fibroblasts (iNOS-F), and endothelial cells (iNOS-E) according to clinicopathologic parameters in cases with gastric carcinoma

**Variables**	**iNOS Score [Mean ± SD (Min.-Max.)]**		
	**iNOS-T**	**iNOS-F**	**iNOS-E**
**Histological type**			
NOS/tubular/papillary	2.6 ± 2.3 (0–6)	4.8 ± 1.7 (2–8)	5.9 ± 2.1 (2–8)
Mucinous/poorly cohesive	4.8 ± 2.2 (2–8)	4.4 ± 1.9 (2–7)	5.9 ± 2.7 (2–8)
**Differentiation**			
Well/moderate	2.6 ± 2.3 (0–6)	4.6 ± 2.2 (0–8)	5.6 ± 2.8 (2–8)
Poorly/signet ring cell	4.8 ± 2.2 (2–8)	4.3 ± 1.3 (0–8)	6.0 ± 1.6 (2–8)
**pN**			
**N0**	**1.3** ± 1.8 (0–5)	2.9 ± 1.3 (2–5)	3.2 ± 1.9 (2–7)
N1	4.1 ± 1.3 (2–6)	5.9 ± 1.6 (4–8)	6.7 ± 1.7 (4–8)
N2	4.1 ± 1.9 (2–8)	4.9 ± 1.2 (3–7)	6.6 ± 1.3 (4–8)
N3	5.9 ± 2.2 (2–8)	5.2 ± 1.2 (3–7)	6.6 ± 1.1 (4–8)
**pT**			
T1	**1.5** ± 1.1 (0–7)	2.4 ± 1.2 (2–7)	3.1 ± 1.7 (2–7)
T2	4.2 ± 1.5 (2–6)	5.4 ± 1.7 (4–8)	6.8 ± 1.8 (4–8)
T3	4.4 ± 1.9 (2–8)	4.9 ± 1.5 (3–7)	6.2 ± 1.7 (4–8)
T4	5.9 ± 2.1 (2–8)	5.1 ± 1.4 (3–7)	6.9 ± 1.2 (4–8)
**TNM stage**			
I	0.0 ± 0.0 (0–0)	3.3 ± 1.5 (2–5)	4.0 ± 2.6 (2–7)
II	3.2 ± 1.8 (0–6)	4.4 ± 1.9 (2–8)	5.0 ± 2.4 (2–8)
III	5.4 ± 3.2 (2–8)	5.3 ± 1.3 (3–7)	6.8 ± 1.0 (4–8)
IV	-	-	-
**Total**	4.**0** ± 2.5 (0–8)	4.0 ± 2.5 (0–8)	5.9 ± 2.0 (2–8)

##### Non-neoplastic gastric tissue

None of the non-neoplastic gastric mucosa samples were positive for iNOS expression in epithelial cells. Of these cases, 20% (n = 4) exhibited focal/weak iNOS expression (score 1–2) in stromal fibroblasts and the vascular endothelium. In all cases, strong iNOS immunoreactivity (score 7–8) was detected in macrophages and neutrophils, which were used as internal positive controls [25-29]. No immunoreaction was observed in negative controls performed without using a primary antibody in any cellular component.

#### D2-40

##### Lymphatic invasion density (LID [nLI/mm^2^])

**Figure 2:**
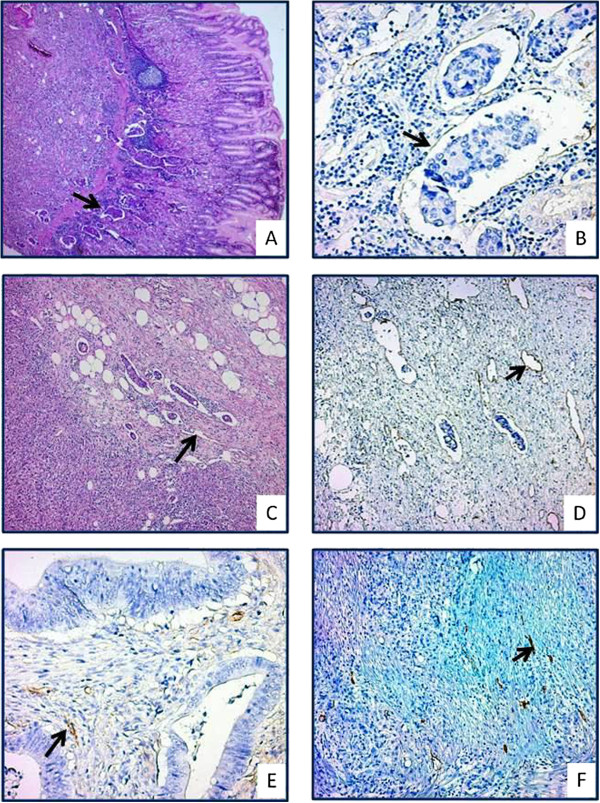
**D2-40 immunoreactivity in gastric carcinoma cases. (A-B)** Tumor cell emboli are observed within lymphatic vessels in a case with gastric carcinoma with diffuse lymphovascular invasion (**A**, H&E; **B**, D2-40). **(C-D)** Dilated lymphatic vessels are displayed in the peritumoral area in a poorly differentiated adenocarcinoma case (**C**, H&E; **D**, D2-40). Intratumoral lymphatic vessels with a narrow lumen are demonstrated in a well differentiated **(E)** and poorly differentiated **(F)** adenocarcinoma cases (**E-F**, D2-40).

D2-40, developed against the oncofetal membrane antigen M2A, is a mAb with an immunoglobulin-G2a (IgG2a) structure that is sensitive to lymphatic endothelial podoplanin. Immunohistochemistry performed using D2-40 revealed lymphatic invasion (LI) in 35 (85.4%) cases (Figure 2A-B). The presence of multiple sites of LI (n > 1) was observed in 100% (n = 41) LI cases (14.39 ± 17.32; range, 1–52). The LID [nLI/mm^2^] of all gastric carcinoma cases was 0.07 ± 0.15 (range, 0.00–0.88).

##### Intratumoral (I-LVD) and peritumoral lymphatic vascular density (P-LVD)

In all cases, lymphatic endothelial cells and/or a lymphatic vascular structure was detected in intratumoral and peritumoral areas that reacted with D2-40. The lymphatic vessels in the intratumoral area were few in number, had a narrow lumen, and were collapsed. In the peritumoral area, however, lymphatic vessels were more prominent, thin walled, irregular, and dilated (Figure 2C-F). The mean P-LVD and I-LVD levels were 13.05 ± 5.67 (median 11.7; range, 5.7–32) and 6.96 ± 3.98 (median 6.0; range, 1–24.3), respectively.

The distribution of LI, LID [nLI/mm^2^], and P-LVD and I-LVD levels by clinicopathological parameter is shown in Table 3.

**Table 3:** Distribution of lymphatic invasion density (LID [nLI/mm2]), peritumoral lymphatic vascular density (P-LVD), and intratumoral lymphatic vascular density (I-LVD) according to clinicopathologic parameters in cases with gastric carcinoma

**Variables**	**Mean ± SD (Min.-Max.)**		
	**LID**	**P-LVD**	**I-LVD**
**Histological type**			
NOS/tubular/papillary	0.06 ± 0.16 (0–0.8)	12.8 ± 2.7 (7.2–15.7)	6.9 ± 4.4 (1–24.3)
Mucinous/poorly cohesive	0.11 ± 0.13 (0–0.8)	13.8 ± 5.4 (5.9–21.6)	6.9 ± 2.6 (3.5–10)
**Differentiation**			
Well/moderate	0.01 ± 0.01 (0–0.03)	10.2 ± 2.7 (7.2–15.7)	5.2 ± 2.1 (1–8.7)
Poorly/signet ring cell	0.10 ± 0.18 (0–0.88)	14.5 ± 6.2 (5.7–32)	6.9 ± 2.6 (3.5–10.2)
**pN**			
**N0**	**0.001** ± 0.003 (0–0.03)	8.1 ± 1.7 (5.9–11.7)	3.5 ± 1.5 (1–5.5)
N1	0.02 ± 0.01 (0–0.04)	8.6 ± 1.9 (5.7–11)	4.8 ± 1.9 (3–8.7)
N2	0.03 ± 0.03 (0–0.02)	12..6 ± 2.5 (9–17.5)	6.8 ± 1.9 (3.5–9.3)
N3	0.18 ± 0.2(0–0.9)	19.1 ± 5.3 (13.7–32)	10.6 ± 4.4 (6–24.3)
**pT**	**0.001** ± 0.003 (0–0.03)	8.1 ± 1.7 (5.9–11.7)	3.5 ± 1.5 (1–5.5)
T1	0.0 ± 0.0 (0–0)	7.9 ± 1.1 (7.2–8.7)	3.9 ± 0.6 (3.4–4.3)
T2	0.0 ± 0.01 (0–0.01)	10.8 ± 7.2 (5.7–16)	6.4 ± 3.2 (4.1–8.7)
T3	0.04 ± 0.05 (0–0.27)	11.8 ± 4.1 (5.9–22.1)	5.9 ± 2.8 (1–11.3)
T4	0.16 ± 0.3 (0–0.88)	19.1 ± 5.3 (13.7–32)	9.6 ± 5.2 (4.3–24.3)
**TNM stage**			
I	0.0 ± 0.0 (0–0)	7.7 ± 0.9 (7.2–8.7)	2.9 ± 1.7 (1–4.3)
II	0.01 ± 0.02 (0.0–0.05)	9.5 ± 2.9 (5.7–16)	4.6 ± 1.9 (1.1–8.7)
III	0.13 ± 0.2 (0.01–0.88)	16.2 ± 5.6 (9–32)	9 ± 4.2 (7.9–11.5)
IV	-	-	-
**Total**	0.07 ± 0.15 (0–0.88)	13.1 ± 5.7 (5.7–32)	6.9 ± 3.9 (1–24.3)

##### Non-neoplastic gastric mucosa

In all non-neoplastic gastric mucosa samples (n = 20; 100%), lymphatic vessels were observed in immunohistochemical studies with D2-40. The mean LVD level in non-neoplastic gastric mucosa was 3.72 ± 1.15.

### Statistical analysis

#### iNOS activity in tumor tissue specimens: correlation with clinicopathological parameters and lymphangiogenesis

##### iNOS expression in tumor cells (iNOS-T)

There was a statistically significant positive correlation between iNOS-T and LID [nLI/mm^2^], I-LVD, and P-LVD. There was a significant difference between iNOS-T and the local inflammatory response, LVI, LI, lymph node involvement, and histological grade (all p-values < 0.05). Tumors with an inflammation score of 3 had a higher iNOS-T score compared with tumors with an inflammation score of ½. Poorly differentiated/signet ring cell carcinoma cases had higher iNOS-T scores than well-/moderately differentiated carcinomas. iNOS-T scores of the cases with negative lymph node status were significantly lower than those with positive lymph node involvement. iNOS-T scores of LVI- and LI-positive cases were significantly higher than LVI and LI negative cases.

##### iNOS expression in tumor-associated stromal fibroblasts (iNOS-F)

There was a positive, weak correlation between iNOS-F and LID [nLI/mm^2^], I-LVD, and iNOS-T. A positive, strong correlation was evident between iNOS-F and iNOS-E. There was a significant difference between pN categories with respect to iNOS-F (pN0 or pN1/2/3). The iNOS-F score in the PN0 category was significantly lower than in other categories (all p-values < 0.05).

##### iNOS activity in endothelial cells (iNOS-E)

There was a positive, weak correlation between iNOS-E and the number of metastatic lymph nodes, nLI, LID [nLI/mm^2^], and P-LVD (all p-values < 0.05).

### D2-40

#### LID (nLI/mm^2^) and LVD: correlation with clinicopathologic parameters

##### LID (nLI/mm^2^)

There was a positive correlation between LID (nLI/mm^2^) and the number of metastatic lymph nodes, I-LVD, P-LVD, and the density of inflammation. There was a statistically significant difference between the histologic grade categories regarding LID (nLI/mm^2^). LID (nLI/mm^2^) was higher in cases with poorly differentiated/signet ring cell carcinoma compared with well-/moderately differentiated carcinomas (all p-values < 0.05).

##### I-LVD and P-LVD

A strong, positive correlation was found between I-LVD and the number of metastatic lymph nodes, tumor diameter, the density of inflammation, and P-LVD. There was a significant difference between histologic grade categories (well-/moderately differentiated or poorly differentiated/signet ring cell) with respect to I-LVD. I-LVD was noted to be significantly higher in the poorly differentiated tumor group (all p-values < 0.05).

There were significant positive correlations between P-LVD and the number of metastatic lymph nodes, tumor diameter, and density of inflammation. There was a significant difference between histologic grade categories with respect to P-LVD. P-LVD was noted to be significantly higher in the poorly differentiated tumor group (all p-values < 0.05).

The mean LVD in the non-neoplastic gastric mucosa was found to be lower than the mean I-LVD (p = 0.003) and P-LVD (p < 0.001) levels of gastric carcinoma cases.

The results of all statistical analyses are shown in Tables 4 and 5.

**Table 4:** Results of statistical analyses between immunohistochemical and clinicopathologic parameters in cases with gastric carcinoma

**Variables**	**iNOS score**			**D2-40**		
	**iNOS-T**	**iNOS-S**	**iNOS-E**	**LID**	**P-LVD**	**I-LVD**
Age*						
*r value*	0.007	0.008	0.07	0.06	0.05	0.01
*p Value*	0.92	0.96	0.86	0.71	0.99	0.85
**Gender (**male or female)**						
*p Value*	0.67	0.93	0.34	0.84	0.20	0.15
**Location of tumor** (proximal or distal) **						
*p Value*	0.53	0.17	0.005	0.95	0.77	0.60
Tumor size (cm)*						
*r value*	0.31	0.06	0.06	0.29	0.53	0.37
*p Value*	0.05	0.70	0.70	0.07	< 0.001	0.01
**Histological type (**NOS/tubular/papillary or mucinous/poorly cohesive) **						
*p Value*	0.46	0.54	0.51	0.14	0.44	0.62
**Differentiation (**Well/moderate or poorly/signet ring cell) **						
*p Value*	0.01	0.748	0.92	0.001	0.001	0.03
**Inflammatory response** (score 1 + 2 or score 3) **						
*p value*	0.01	0.89	0.51	0.02	0.001	0.01
**LVI [H&E](positive or negative)** **						
*p Value*	0.002	0.03	0.01	0.001	0.001	< 0.001
**LI [D2-40](positive or negative)** **						
*p Value*	0.002	0.02	0.01	< 0.001	0.001	< 0.001
nLI*						
*r value*	0.64	0.37	0.44	0.98	0.65	0.63
*p Value*	< 0.001	0.017	0.004	< 0.001	< 0.001	< 0.001
nNm*						
*r value*	0.61	0.30	0.40	0.83	0.86	0.78
*p Value*	< 0.001	0.05	0.009	< 0.001	< 0.001	< 0.001
**pN (N0 or N1/2/3)** **						
*p Value*	0.001	0.003	0.023	< 0.001	< 0.001	< 0.001
**pT** (T1 + 2 orT3 + 4) **						
*p Value*	0.05	0.70	0.70	0.07	< 0.001	0.01
**TNM stage** (I + II or III + IV) **						
*p Value*	0.0002	0.067	0.02	< 0.001	< 0.001	< 0.001

* Pearson’s correlation analysis/Spearman’s correlation analysis and **Independent sample *t* test/the Mann–Whitney U tests were used.

**Table 5:** Results of statistical analyses* of the relationship of iNOS scores at different cellular localizations and lymphatic invasion density (LID [nLI/mm2]), peritumoral lymphatic vascular density (P-LVD), and intratumoral lymphatic vascular density (I-LVD)

**Variables**	**iNOS-T**	**iNOS-F**	**iNOS-E**	**LID**	**P-LVD**	**I-LVD**
**LID**						
*r value*	0.64	0.39	0.45	-	0.65	0.63
*p value*	0.001	0.013	0.004	-	0.001	0.001
**P-LVD**						
*r value*	0.56	0.27	0.35	0.65	-	0.84
*p value*	0.001	0.08	0.02	0.001	-	0.001
**I-LVD**						
*r value*	0.59	0.32	0.25	0.63	0.84	-
*p value*	0.001	0.04	0.11	0.001	0.001	-

* Pearson’s correlation analysis/Spearman’s correlation analysis were used.

## Discussion

The lymphatic system serves as the primary route for metastatic spread of gastric carcinomas, and the presence and extent of lymph node involvement are the principal parameters affecting survival [30-32]. Growth factors (e.g., VEGF-C and VEGF-D) associated with lymphangiogenesis increase in malignant melanoma and breast, lung, and colon carcinoma [30-33]. Many studies have suggested that lymphangiogenesis is a more reliable parameter than angiogenesis in predicting tumor progression [30-34]. Despite all these data, how gastric tumor cells reach the potential to metastasize via lymphatic vessels and the biological role of tumor-associated lymphangiogenesis in the development of nodal metastases remain unclear.

Various methods are used to determine lymphangiogenic activity. Some studies have supported that the rate of LEC proliferation reflects lymphangiogenic activity [34,35]. In most studies, LVD detection using lymphatic vessel endothelium-specific monoclonal antibodies (mAbs) is a reliable indicator of lymphangiogenesis [30-39]. Therefore, we considered lymphatic endothelial cells to be independent of their proliferative activity. Using lymphatic endothelium-specific markers is a reliable method in the determination of lymphatic vessels and real LI in tumor tissue. For this purpose, many lymphatic endothelium-specific mAbs (e.g., anti-VEGFR-3, anti-LYVE-1, anti-Prox-1, anti-LyP-1, and anti-Nrp2) have been produced. D2-40 is the preferred mAb for investigating intratumoral and peritumoral lymphatics because it is more convenient and more sensitive than other lymphatic markers [40]. In our study, we used podoplanin (clone D2-40) to determine LI and LVD; its reliability has been proven in previous studies [33-36,40].

The microvascular density (MVD) method used by Weidner et al. for the determination of tumor angiogenesis is the most commonly used for LVD determination [22]. It has been reported that the mean vessel number in three areas where vascular structures are most dense (hot spot) is sufficient to determine tumor angiogenesis [23]. However, tumor-associated lymphatic vessels are fewer and more dispersed than blood vessels. Thus, assessment of an area as large as possible for the determination of LVD will be a more reliable marker of lymphangiogenic activity [24,26,35]. Thus, the mean number of lymphatic vessels in 10 hot spots was used as the LVD in this study.

In our study, varying numbers of lymphatic endothelium/vessels were present in both intratumoral and peritumoral areas in all cases with gastric carcinoma. Lymphatic vessels in intratumoral areas were of immature appearance and collapsed. Lymphatic vessels were concentrated in peritumoral areas. Peritumoral lymphatic vessels had thin walls, irregular shapes, and dilated appearances. It has been argued that compression by tumor cells and oncotic pressure increase in intratumoral areas, causing lymphatic vessels to be few in number and have a narrow lumen [41]. The morphological findings of our study support this view. In agreement with previous studies, we found that P-LVD was significantly higher than I-LVD. A positive correlation was found between I-LVD and P-LVD [26,42-46]. This finding suggests that lymphangiogenic growth factors expressed from the tumor microenvironment affect intratumoral and peritumoral areas to different extents; however, the lymphangiogenic activity at both localizations is closely interrelated.

Lymphatic vessels are highly dynamic structures that intimately interact with their surrounding microenvironment. They have a profound influence on the immune system and therefore can manipulate inflammatory processes. Inflammation is a major cause of adulthood lymphangiogenesis and lymphovascular remodeling [41]. Our study demonstrated that lymphatic vessel density in tumor tissue (I-LVD and P-LVD) was positively correlated with the extent of the local inflammatory response. Our findings suggest that the immune response to the tumor is a lymphangiogenesis-inducing factor and may play a role in the formation of tumor lymphatic metastases. Cytokines and the metabolic load increase during inflammation may cause this phenomenon [41].

P-LVD has been shown to be associated with poor prognosis and short survival in breast, colon, and lung cancers [26,31,44,45]. However, the effect of I-LVD on tumor progression remains a controversial issue [26-34]. Our study demonstrated that both I-LVD and P-LVD were positively correlated with LI, LID, the presence of lymph node metastasis, and the number of metastatic lymph nodes. Those findings show that an increase in lymphangiogenic activity accelerates the development of nodal metastases by facilitating the transport of tumor cells to lymphatic vessels. Furthermore, our findings suggest that both I-LVD and P-LVD can be used for the prediction of lymphatic spread of the tumor.

The genesis of lymph node metastasis in tumors is a complex and multifactorial process. Invasion of lymphatic vessels at the primary tumor focus by tumor cells is the first and primary step. However, LI cannot be regarded as the sole indicator of lymph node metastasis because of the antitumor defense mechanisms of the host and tumor-associated lymphatic vessels containing anatomically/functionally abnormal features. An increased LI number in tumor tissue will increase the likelihood of metastasis by causing more tumor cells to enter the circulation [16,25,26,38,39]. Therefore, we assessed tumor cell entry into the lymphatic circulation using two parameters: nLI and LID (nLI/mm^2^). We detected a significant positive relationship between nLI, and LID and the presence of lymph node metastasis, and the number of metastatic lymph nodes. Our results suggest that the presence of multiple lymphatic invasion sites at the primary tumor focus increases the chance of tumor cells metastasizing to lymph nodes. In addition, LID may be a more reliable marker for predicting lymph node metastasis than LVI or LI.

It has been demonstrated *in vitro* and *in vivo* that NO molecules exert significant effects on tumor lymphangiogenesis and lymphatic spread in addition to tumor angiogenesis. NO induces the proliferation of lymphatic endothelial cells and prolongs their survival by increasing lymphangiogenic growth factors (e.g., VEGF-C/VEGF-D) and VEGFR-2/VEGFR-3 expression in tumor-associated lymphatic vessels [8]. It has been determined that the increase in NOS activity in tumor tissue is positively correlated with lymphatic metastasis in head and neck, breast, thyroid, and gall bladder cancers and malignant melanomas [3-8]. Lahdenranta et al. reported that blocking NOS activity in fibrosarcomas prevented peritumoral lymphatic hyperplasia and tumor cell spread to lymph nodes [6].

Studies of iNOS expression in gastric carcinomas frequently indicate a relationship between NO and angiogenesis. Few studies have examined the relationship between NOS and lymphangiogenesis in gastric carcinomas. Wang et al. determined that, compared with normal gastric tissue, gastric carcinomas exhibited greater iNOS expression. In that study, it was determined that iNOS expression in tumor cells is a reliable marker of the iNOS mRNA level [11]. Koh et al. reported that iNOS expression was closely related to the loss of differentiation in tumor cells and elevated levels of pro-inflammatory cytokines (e.g., TNFα) [19]. Li et al. identified moderate-to-high iNOS expression in 62% of gastric carcinomas. These researchers found a significant correlation between iNOS expression in tumor cells and tumor size, invasion depth, lymph node involvement, and TNM stage [18]. Zhang et al. reported that iNOS expression was an independently associated with survival [5].

We did not identify iNOS expression in non-neoplastic gastric epithelial cells. We found varying degrees of iNOS expression in tumor cells in 87.8% of gastric carcinoma cases. We also identified a significant correlation between iNOS expression in tumor cells and inflammation density, loss of differentiation, and parameters related to lymphatic tumor spread/lymphangiogenesis. We found that iNOS expression was more prominent, particularly in EMT-like dedifferentiation areas with loss of cohesion and an invasive phenotype. Our results suggest that tumor cells are a principal source of iNOS, and an increase in iNOS activity has the potential to modulate lymphangiogenic activity in gastric carcinomas. Furthermore, our results indicate that tumor cells with a less differentiated/invasive phenotype express more iNOS, and that this increased iNOS expression contributes to metastatic spread. Similar to Koh et al. [19], we showed that iNOS expression and the inflammatory response had a positive correlation. Our findings suggest that the increase in iNOS expression in tumor cells is a common factor that induces both a tumor-associated inflammatory response and lymphangiogenesis.

iNOS is expressed primarily by macrophages and neutrophils [2-8,29]. In neoplastic tissues, tumor cells are a primary source of iNOS. Thomsen et al. showed that stromal fibroblasts and endothelial cells expressed iNOS, and stromal iNOS expression was correlated with tumor grade in breast carcinomas [29]. We assessed iNOS expression in various cellular components of gastric carcinomas separately, and found that iNOS expression in tumor cells and iNOS expression in stromal cells were positively correlated. Our findings indicate that the NO level in the microenvironment of the tumor may be elevated by iNOS originating from various cellular components. In addition, we also found that iNOS expression in the tumor stroma was related to tumor lymphangiogenesis and lymphatic spread. However, compared with that originating from tumor cells, iNOS expression from tumor-associated stroma appears to play a less important biological role.

## Conclusions

In conclusion, we found that both intratumoral and peritumoral LVD were closely related to lymph node metastasis, the most important parameter of the biological behavior of a tumor, in gastric carcinomas. We also found that iNOS may be expressed by different cellular components in tumor tissues; indeed, iNOS expressed by tumor cells may play an important role in the pathogenesis and progression of gastric carcinomas. Our finding of an increase in the iNOS level in EMT-like dedifferentiation areas, reflecting transformation of tumor cells into an invasive and a metastatic phenotype, supports this result. In addition, our findings indicate the potential of an iNOS-mediated NO increase to modulate lymphangiogenesis and lymphatic spread in gastric carcinomas. Therefore, development of novel agents that inhibit iNOS activity and NO signal conduction may represent an alternative treatment of gastric carcinomas.

## Consent

Written informed consent was obtained from the patient for the publication of this report and any accompanying images.

## Abbreviations

H&E: Hematoxylin and eosin; I-LVD: Intratumoral lymphatic vascular density; iNOS: Inducible nitric oxide synthases; iNOS-T: iNOS expression of tumor cells; iNOS-E: iNOS expression of endothelial cells; iNOS-F: iNOS expression in tumor-associated stromal fibroblasts; LECs: Lymphatic endothelial cells; LI: Lymphatic invasion; LID: Lymphatic invasion density; LVD: Lymphatic vascular density; LVI: Lymphovascular invasion; P-LVD: Peritumoral lymphatic vascular density; NO: Nitric oxide; NOS: Nitric oxide synthases; mAbs: Monoclonal antibodies; nLI: Number of lymphatic invasion; nNm: Number of metastatic lymph nodes; VEGF: Vascular endothelial growth factor.

## Competing interests

The authors declare that they have no competing interests.

## Authors’ contributions

NK conducted the study design, performed microscopic evaluation, and drafted the manuscript. NOK participated in the design of the study and helped to draft the manuscript. DY, GG and TK participated in the design of the study and performed the selection of appropriate cases and data collection and helped to draft the manuscript. FK participated in the design of the study and performed statistical analyses. All authors read and approved the final manuscript.
